# Design of Poly(lactic-*co*-glycolic Acid) (PLGA) Nanoparticles for Vaginal Co-Delivery of Griffithsin and Dapivirine and Their Synergistic Effect for HIV Prophylaxis

**DOI:** 10.3390/pharmaceutics11040184

**Published:** 2019-04-16

**Authors:** Haitao Yang, Jing Li, Sravan Kumar Patel, Kenneth E. Palmer, Brid Devlin, Lisa C. Rohan

**Affiliations:** 1 Department of Pharmaceutical Sciences, School of Pharmacy, University of Pittsburgh, Pittsburgh, PA 15261, USA; hyang96@its.jnj.com (H.Y.); jil132@pitt.edu (J.L.); patels10@mwri.magee.edu (S.K.P.); 2 Magee-Womens Research Institute, Pittsburgh, PA 15213, USA; 3 Center for Predictive Medicine and Department of Pharmacology and Toxicology, University of Louisville School of Medicine, Louisville, KY 40202, USA; kenneth.palmer@louisville.edu; 4 International Partnership for Microbicides, Silver Spring, MD 20910, USA; bdevlin@ipmglobal.org; 5 Department of Obstetrics, Gynecology and Reproductive Sciences, University of Pittsburgh, Pittsburgh, PA 15261, USA

**Keywords:** microbicides, HIV, combination ARVs, sustained release, synergism, PLGA nanoparticles, co-delivery

## Abstract

Long-acting topical products for pre-exposure prophylaxis (PrEP) that combine antiretrovirals (ARVs) inhibiting initial stages of infection are highly promising for prevention of HIV sexual transmission. We fabricated core-shell poly(lactide-*co*-glycolide) (PLGA) nanoparticles, loaded with two potent ARVs, griffithsin (GRFT) and dapivirine (DPV), having different physicochemical properties and specifically targeting the fusion and reverse transcription steps of HIV replication, as a potential long-acting microbicide product. The nanoparticles were evaluated for particle size and zeta potential, drug release, cytotoxicity, cellular uptake and in vitro bioactivity. PLGA nanoparticles, with diameter around 180–200 nm, successfully encapsulated GRFT (45% of initially added) and DPV (70%). Both drugs showed a biphasic release with initial burst phase followed by a sustained release phase. GRFT and DPV nanoparticles were non-toxic and maintained bioactivity (IC_50_ values of 0.5 nM and 4.7 nM, respectively) in a cell-based assay. The combination of drugs in both unformulated and encapsulated in nanoparticles showed strong synergistic drug activity at 1:1 ratio of IC_50_ values. This is the first study to co-deliver a protein (GRFT) and a hydrophobic small molecule (DPV) in PLGA nanoparticles as microbicides. Our findings demonstrate that the combination of GRFT and DPV in nanoparticles is highly potent and possess properties critical to the design of a sustained release microbicide.

## 1. Introduction

Human immunodeficiency virus (HIV) infection is one of the world’s most serious health challenges, and the development of means to prevent its spread is urgently needed. By the end of 2017, approximately 36.9 million people were living with HIV, with 1.8 million more becoming newly infected globally [1]. Women, especially young (15–24 years) and adolescent (10–19 years), are disproportionately affected by HIV due to social, cultural, economic, and biological vulnerabilities [2,3]. Sexual transmission is the principal cause of new HIV-1 infections in women in the U.S. and worldwide [2].

To protect women from this global epidemic, it is critical to develop effective biomedical interventions for HIV/AIDS prevention and treatment. However, there is no effective vaccine available. In addition, the low instance of condom use by men and other social factors could significantly increase HIV-1 infection in women [4,5]. With these considerations in mind, oral or topical pre-exposure prophylaxis (PrEP) with antiretroviral (ARV) drugs could be the most promising avenue for preventing sexually transmitted HIV infection. Topical PrEP products could be applied by women in the vaginal or rectal tract before sexual intercourse to inactivate pathogens, including HIV, thus providing a female-controlled strategy against HIV-1 infection [6,7,8]. Rectal-specific topical PrEP products are equally applicable to men who have sex with men (MSM).

HIV-specific ARV drugs that can target specific steps in HIV’s life cycle and then effectively inhibit its replication have been selected in the development of current microbicides [9]. To reach maximum protection effect of microbicides, ARVs inhibiting early stage of HIV replication can be beneficial. A combination strategy that includes multiple ARVs targeting different steps of HIV infection lifecycle could potentially improve the antiretroviral activity of each product with synergistic effect as a viable PrEP product for preventing acquisition of HIV-1. It has been proven that combination ARV (cARV) therapy can increase the efficacy of each drug in clinic, reduce resistance, decrease the risk of both HIV disease progression and death, reduce doses and cost, and improve patient adherence and compliance [10].

Dapivirine (DPV) is a diarylaminopyrimidine compound with high activity against HIV-1, and it belongs to the family of non-nucleoside reverse transcriptase inhibitors (NNRTIs). It is extremely potent in inhibiting both wild-type and mutant HIV-1 in vitro [11]. No vaginal or systemic toxicity of DPV was observed in sheep, indicating a desirable safety profile of DPV as a microbicide candidate [12]. In an intravaginal ring dosage form, DPV has shown great promise in Phase III clinical trials, ASPIRE and The Ring study [13,14]. Another promising molecule is Griffithsin (GRFT), a 121–amino acid and13-kDa-molecular-mass lectin that was originally isolated from the red alga *Griffithsia* sp. [15]. Binding to the mannose-rich glycans gp120 and gp41, GRFT inhibits HIV-1 gp120 and exposes the CD4 binding site [16,17]. GRFT is shown to exhibit high potency in inhibition of both X4- and R5-tropic HIV-1 virus at subnanomolar concentration, with high stability in cervical/vaginal lavage fluid [18]. It has been shown to be safe and tolerable in subcutaneous treatment in both guinea pigs and mice at 10 mg/kg and in rabbit vaginal irritation studies [19,20]. Furthermore, our previous investigation of the effect of GRFT rectal gel formulations on proteome and microbiome in non-human primates was supportive of their safety [21]. Importantly, GRFT has shown strong synergistic activity when combined with nucleoside reverse transcriptase inhibitor (NRTI), NNRTI and fusion inhibitor compounds, such as tenofovir, maraviroc, and enfuvirtide in vitro [22]. Furthermore, the broad-spectrum anti-HIV activity and unique safety profile of GRFT and DPV support their development as combined topical microbicides. Therefore, we hypothesized that GRFT will display strong synergistic activity with DPV when combined in application as a microbicide product.

The extreme opposite properties of these two molecules greatly hinder the development of a formulation for the simultaneous delivery of both drugs. The proteinaceous nature of GRFT and quick tissue elimination and low water solubility of DPV, pose limitations to their topical microbicide potential [23]. Additionally, developing a long-acting drug delivery system, which is more favorable for patient adherence of the PrEP product [24,25], will meet with additional challenges. Therefore, our goal was to develop a novel drug delivery system that overcomes formulation limitations and co-delivers GRFT and DPV with a sustained release profile to solve the clinical need in microbicides.

Nanoparticle drug delivery systems not only provide the sustained or controlled delivery of APIs, but also improve drug solubility, protect drug payloads, and enhance mucosal drug permeability [26]. With these advantages, nanoparticle delivery systems have been explored in the design of vaginal microbicides [27,28,29,30,31]. Polymeric nanoparticles can provide controlled or sustained release profiles for the payloads. Poly(ethylene oxide) (PEO) modified poly(caprolactone) (PCL) nanoparticles have been developed for the delivery of DPV as an alternative vaginal microbicide [31] and showed enhanced mucosal penetration and improved local pharmacokinetic profiles of DPV [29]. Nanoparticles made from PEO modified poly(lactic-*co*-glycolic acid) (PLGA) were applied to deliver rilpivirine and demonstrated improved vaginal tissue concentration [32]. Previous studies have utilized nanoparticles for delivery of DPV [33]. Here, we used a PLGA-based nanoparticle delivery system to deliver GRFT/DPV simultaneously in a sustained release pattern. Furthermore, encapsulation of GRFT and DPV in PLGA nanoparticles overcomes the limitations of poor tissue permeability and low aqueous solubility respectively. PLGA is one of the most widely used biodegradable polymers for drug delivery applications due to its superior biocompatibility and biodegradability [34]. It is an FDA-approved polymer that has been widely used for the delivery of proteins/peptides and small molecules [35,36,37,38]. Importantly, PLGA nanoparticles were found to have no immunogenicity in vivo, which is critical for the safety of an anti-HIV drug delivery system [39,40,41,42]. Additionally, PLGA nanoparticles have been studied in the delivery of several ARV drugs for HIV/AIDS treatment and prevention [38,43,44,45,46]. However, to our knowledge, no studies have yet been reported to deliver both a macromolecule and a small hydrophobic molecule simultaneously in one nanoparticle system for PrEP. This attempt on the co-delivery of a macromolecule and a small hydrophobic molecule with PLGA nanoparticles will have implications for the development of future microbicides.

In this study, we investigated the ability of PLGA nanoparticles to deliver both GRFT and DPV simultaneously and their anti-HIV efficacy in combined application as ARVs. We show that GRFT and DPV can be individually and simultaneously fabricated into biodegradable PLGA nanoparticles with a high encapsulation efficiency. The ARV-containing nanoparticles were nontoxic in cell culture. We also observed synergistic effects in the combined application of GRFT and DPV. Collectively, our data show that core-shell type PLGA nanoparticles are a promising strategy for delivering multiple ARV drugs with extreme physicochemical diversity. Our results support a viable nanoparticle platform for the delivery of multi-ARV combinations for sustained HIV-1 prevention.

## 2. Materials and Methods

### 2.1. Materials

PLGA with lactic acid to glycolic acid ratios of 50:50 was purchased from Sigma-Aldrich (Resomer^®^ RG 502 H, MW, 30 kDa; St. Louis, MO, USA). Cell culture reagents were obtained from GIBCO, Invitrogen by Life Sciences, Inc. (Lenexa, KS, USA). DPV was obtained from International Partnership for Microbicides (Silver Spring, MD, USA). Recombinant GRFT (~13 kDa) was kindly provided by Kentucky Bioprocessing, LLC (Owensboro, KY, USA). The protein was supplied in a solution of phosphate buffered saline. Phosphate buffered saline 10× molecular biology grade was purchased from Mediatech, Inc. (Manassas, VA, USA). All other reagents used for nanoparticle formulation were purchased from Thermo Fisher Scientific (Pittsburgh, PA, USA).

### 2.2. Methods

#### 2.2.1. Fabrication of ARV-Loaded Nanoparticles

**Figure 1:**
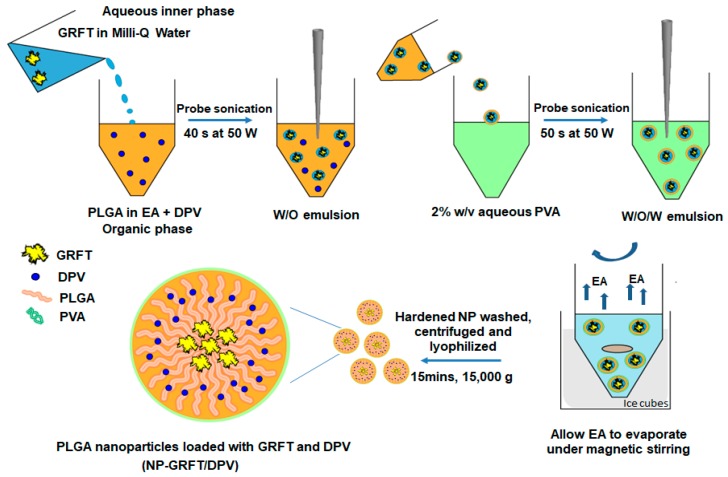
Schematic representation of DPV and GRFT-loaded PLGA nanoparticle preparation by double emulsion-solvent evaporation method.

The ARV loaded nanoparticles were prepared by using double emulsion-solvent evaporation technique as shown in Figure 1. Blank nanoparticles (vehicle control) were prepared using this technique at ambient temperature, as previously described, with some modifications [38]. Briefly, 100 µL Milli-Q water as inner water phase was added into a solution of PLGA (20 mg) in ethylacetate (EA) (1 mL). Then, the primary water-in-oil (W/O) emulsion was obtained by homogenization using a 6-mm diameter Vibra-Cell probe sonicator (Sonics and Materials, Newton, CT, USA) for 40 s at 50 W. The primary W/O emulsion was then added into 2 mL of 2% *w*/*v* aqueous solution of polyvinyl alcohol (PVA) with sonication for 50 s at 50 W to form the secondary water-in-oil-in-water (W/O/W) emulsion. The W/O/W nanoparticle solution was then diluted with 10 mL of Milli-Q water under magnetic stirring for 4 h in an ice water bath to allow EA to evaporate. The hardened nanoparticles were washed with deionized water three times by centrifugation for 15 min at 15,000× *g* (Sorvall Ultra 80, Waltham, MA, USA). Nanoparticles were then re-suspended in 1 mL of Milli-Q water after removing the PVA supernatant and were then lyophilized overnight (approximately 12 h) under vacuum at 0.120 mbar and at −50 °C using a FreeZone 6 lyophilizer (Labconco, Kansas City, MO, USA). Nanoparticles loaded with GRFT, GRFT/DPV, and DPV were fabricated similar to the blank nanoparticles. GRFT (50 µL, 10 mg/mL) was dissolved in the inner water phase for GRFT nanoparticle preparation. DPV was dissolved in EA (0.033 mg/mL) with PLGA polymer in DPV nanoparticles. GRFT/DPV nanoparticles were prepared by dissolving GRFT in inner water phase and DPV in EA. GRFT and DPV loading levels were kept the same in each preparation unless noted otherwise. The lyophilized nanoparticles were stored in aliquots in glass vials at 4 °C until use. To prepare fluorescent nanoparticles, fluorescein isothiocyanate–dextran 70 (FITC-dextran, MW 70,000; Sigma LLC, St. Louis, MO, USA) and Nile red (Sigma LLC) were added to inner phase and EA respectively during the manufacture of blank NPs.

#### 2.2.2. Characterization of Nanoparticles

Size and zeta potential of the fabricated nanoparticles were determined using a Zetasizer Nano ZS90 (Malvern Instruments, Malvern, UK). Size and morphology of the nanoparticles were also confirmed by Transmission Electron Microscope (TEM), visualized with a JEM 1011 (JEOL, Sheboygan, WI, USA) scanning electron microscope. Samples of nanoparticles were negatively stained with ammonium phosphomolybdate and imaged using an 80 kV electron beam at the Center for Biologic Imaging of the University of Pittsburgh. The zeta potential of the PLGA nanoparticles, both drug-free and drug-loaded, in Milli-Q water, was measured using the zeta potential analysis mode in the Zetasizer.

#### 2.2.3. Drug Loading

The amount of GRFT and DPV encapsulated in the nanoparticles was determined by analyzing the GRFT and DPV content in the supernatant after centrifugation by high-performance liquid chromatography (HPLC) to quantify encapsulation efficiency (Equation (1)). For GRFT analysis, an HPLC system (Waters Corporation, Milford, MA, USA) equipped with an auto injector (model 717), a quaternary pump (model 600), an ultraviolet detector (model 2487) at 280 nm, and a multi λ fluorescence detector (model 2475) at 370 nm was used. Separation of GRFT was achieved by using a Jupiter 5 µ 300 Å (250 × 4.6 mm) column (Phenomenex, Torrance, CA, USA) protected by a guard cartridge Jupiter C18 (4.0 × 3 mm). A gradient consisting of mobile phase A (0.1% trifluoroacetic acid [TFA] in Milli-Q water), and mobile Phase B (0.1% TFA in acetonitrile), at a flow rate of 1.0 mL/min, was used. Retention time of GRFT was approximately 16 min, and the total run time was 30 min. Empower Pro 3 software (Waters Corp., Milford, MA) was used to control the HPLC system.

DPV was assayed by using an Acquity ultra-performance liquid chromatography (UPLC) system H-class (Waters Corp., Milford, MA). Separation of DPV was achieved by using an Acquity UPLC BEH C18 (1.7 µm, 2.1 × 50 mm) column (Waters Corp., Milford, MA). A gradient consisting of mobile phase A (0.1% TFA in Milli-Q water) and mobile Phase B (0.1% TFA in acetonitrile), at a flow rate of 0.25 mL/min, was used. Retention time of DPV was approximately 8.5 min, and the total run time was 15 min. Empower Pro 3 software was used to control the UPLC system. Drug encapsulation efficiency is calculated by following equation as described by Papadimitriou et al. [47]:

(1) $$\begin{array}{ll} \mathrm{Drug}\;\mathrm{encapsulation}\;\mathrm{efficiency}\;\left(\%\right) & =\frac{\mathrm{weight}\;\mathrm{of}\;\mathrm{drug}\;\mathrm{in}\;\mathrm{nanoparticles}}{\mathrm{weight}\;\mathrm{of}\;\mathrm{drug}\;\mathrm{fed}\;\mathrm{initially}}\\ & =\frac{\mathrm{weight}\;\mathrm{of}\;\mathrm{drug}\;\mathrm{fed}\;\mathrm{initially}\;\mathrm{weight}\;\mathrm{of}\;\mathrm{drug}\;\mathrm{in}\;\mathrm{supernantant}\;}{\mathrm{weight}\;\mathrm{of}\;\mathrm{drug}\;\mathrm{fed}\;\mathrm{initially}} \end{array}$$

#### 2.2.4. In Vitro Release of ARVs from PLGA Nanoparticles

To determine the extent and rate of GRFT and DPV released from the nanoparticles, an in vitro release study was conducted over a 7-day period. In this study, vaginal fluid simulant (VFS, pH 4.5) was selected as dissolution medium to evaluate drug release from the fabricated nanoparticles because of its physiological relevance to the cervicovaginal fluid [48]. ARV-loaded nanoparticles were dispersed in 3 mL of VFS, with continuous shaking, at a temperature of 37 °C. At regular intervals, the nanoparticles were isolated via centrifugation (12,000× *g*, 4 °C, 30 min), and the entire VFS solution was decanted for analysis using chromatographic methods describe above. The nanoparticles were then re-suspended in fresh VFS and returned to the in vitro release set-up.

To determine the impact of medium and pH on drug release, in vitro release studies were also performed in 1 M phosphate-buffered saline (PBS) at pH 7.4 and 1 M PBS at pH 4.5 (1 M PBS adjusted to pH 4.5 with 10% hydrochloric acid). The amount of GRFT and DPV released into the release media was determined by HPLC or UPLC methodology as previously detailed.

#### 2.2.5. Anti-HIV-1 Activity and Cellular Viability Assay of ARVs

The cytotoxicity and anti-HIV-1 activity of free and nanoparticle-formulated ARVs against HIV-1_BaL_ was determined in TZM-bl cells by luciferase quantification of cell lysates [49,50,51]. The HIV-1_BaL_ and TZM-bl indicator cell line were kindly provided by Dr. Charlene Dezzutti of Magee-Womens Research Institute. TZM-bl is a HeLa cell line derivation that stably expresses high levels (% positive cells) of CD4 (32%), CCR5 (82%) and CXCR4 (85%) [52]. The cells contain HIV-1 Tat-regulated reporter genes for firefly luciferase and β-galactosidase for quantitative analysis of HIV-1, simian immunodeficiency virus (SIV), and simian/human immunodeficiency virus (SHIV) infection [53]. The evaluation of cytotoxicity and anti-HIV-1 activity were conducted following methods previously described [54]. The cells were regularly cultured in Dulbecco’s Modified Eagle Medium (DMEM) with 10% fetal bovine serum (FBS), 100 U/mL penicillin, 100 μg/mL streptomycin, and 1% 200 mM L-glutamine at 37 °C in 5% CO_2_ atmosphere.

The TZM-bl cells were seeded into a 96-well clear-view plate, at a concentration of 5 × 10^4^ cells per well, in 100 μL of DMEM medium (10% FBS) and allowed to adhere for 24 h at 37 °C [54]. Identical, but separate, plates were set up to measure efficacy and cellular toxicity. 100 μL of medium was removed and replaced with 100 μL of DMEM dilutions of test articles i.e., each drug and ARV-loaded nanoparticles (NP-ARVs). Cells exposed to media without test articles was used as a control. For toxicity testing, additional 100 μL of medium (without test articles) was added to each well to bring the total volume to 200 μL. The next day, 100 μL of medium was removed and replaced with 100 μL of CellTiter-Glo^®^ (Promega, Madison, WI, USA) and the luminescence measured using SpectraMax M3 plate reader (Molecular Devices, Sunnyvale, CA, USA). Viability was determined on the basis of deviations from the cell-only control and presented as the percentage viability ± SD (standard deviation).

For efficacy testing, following addition of media with and without test articles, 100 μL of medium containing HIV-1 was added to each well to reach a total volume in each well of 200 μL. HIV-1_BaL_ was added at an approximate TCID_50_ (50% tissue culture infectious dose) of 3000 per well in the presence of 40 μg/mL of Diethylaminoethyl cellulose- Dextran (DEAE-Dextran, Sigma, St. Louis, MO, USA). After 48 h, 100 μL of medium was removed and replaced with 100 μL of Bright-Glo™ (Promega, Madison, WI, USA) and the luminescence measured as mentioned above. Inhibition of HIV infection was determined on the basis of luminescence deviations from the HIV-1-only control and presented as the percentage inhibition ± SEM. Untreated wells with cells only served as the negative infectivity control (100% inhibition), while wells with cells and HIV-1 served as positive infectivity control (0% inhibition). The percent inhibition was calculated for all test and control cultures to determine the 50% inhibition concentration (IC_50_) value of each drug. The IC_50_ values of NP-ARVs were calculated via GraphPad Prism (V 5.02) software, using drug concentrations that corresponded to the actual drug loading determined by HPLC.

#### 2.2.6. Combination Effects

The combined anti-HIV-1 activity of the dual-drug combination was evaluated by using the median-effect analysis described by Chou and Talalay [55]. Briefly, median values (IC_50_) of each drug were obtained using the TZM-bl antiviral activity assay as described in the previous section. The drugs were then mixed according to the ratio of their individual IC_50_ values (1:8, due to the molecular weight difference between DPV and GRFT). For NP-ARVs, amounts of the individual compounds used in combinations were determined by measured drug loading. The drug mixtures were then serially diluted, and IC_50_ values were determined with the TZM-bl assay. Combination effects of ARVs were evaluated by (1) comparing HIV-1 inhibition between the combination and each individual ARV; and (2) identifying combination indices (CI) to quantify drug synergy, using CompuSyn software (ComboSyn, Inc., Paramus, NJ, USA). The CI of drug combination was plotted as a function of the fractional inhibition (Fa), by computer simulation, from Fa = 0.10 to 0.95. In this analysis, the combined effect at the 50% fractional inhibition (CI_50_) and 90% fractional inhibition (CI_90_) were reported as synergistic, additive, or antagonistic when CI < 1, = 1, or >1, respectively.

#### 2.2.7. Cellular Uptake Assay

The mouse macrophage cell line RAW264.7 (ATCC, Manassas, VA, USA) was maintained in DMEM culture medium with 10% FBS, 100 U/mL penicillin, 100 μg/mL streptomycin, and 1% 200 mM L-glutamine at 37 °C in 5% CO_2_ atmosphere. RAW264.7 cells were cultured on a 1-cm diameter coverslip pre-coated with polylysine (BD BioCoat, BD Biosciences, Bedford, MA, USA) in DMEM culture medium for 48 h before uptake assay. Fluorescent dye-loaded NPs were added to the cells at 2.5% (*w*/*v*) of the total 200 µL of cell culture media for 4 h at 37 °C. NPs were then washed from cells with PBS for 4 times. The coverslips with cells grown on them were first stained with 4′,6-diamidino-2-phenylindole (DAPI, Molecular Probes, and Eugene) in PBS in order to visualize the nuclei and were then mounted on glass slides for fluorescent imaging. The fluorescent images were obtained by ApoTome Confocal Microscope Observer Z1 (Carl Zeiss Microscopy, Zaventem, Belgium). Cell images were taken at 358/461 nm for DAPI, 495/519 nm for FITC-dextran, and 552/636 nm for Nile red.

#### 2.2.8. Statistical Analyses

All differences were evaluated by Student’s t-test or ANOVA analysis. *p* < 0.05 was considered statistically significant. Drug release profiles were analyzed using DD Solver, an Excel add-in package [56]. All error bars represent standard deviations unless otherwise noted.

## 3. Results

### 3.1. PLGA Nanoparticle Characterization

**Figure 2:**
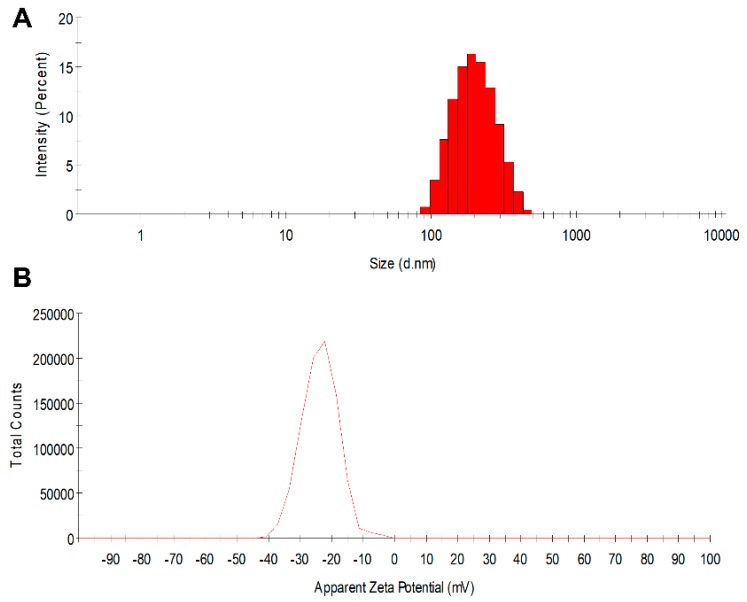
Characterization of PLGA nanoparticles loaded with GRFT and DPV. (**A**) Size and (**B**) zeta potential distribution graphs of nanoparticles encapsulated with anti-HIV drugs GRFT and DPV (NP-DPV/GRFT).

In this study, PLGA core-shell nanoparticles were successfully developed that simultaneously encapsulate and deliver both a protein drug and a small-molecule hydrophobic drug without compromising the effectiveness of either. The manufactured PLGA nanoparticles are near-spherical in shape, possess high drug-loading capability, and are of a reproducible size. It has been reported that the duration and intensity of sonication can significantly affect the size of the nanoparticles produced using double-emulsion method [57,58]. The targeted particle size and distribution can be obtained by optimizing sonication intensity and time within a given system. With the sonication parameters described in the Methods section, the diameters of the fabricated nanoparticles were confined to a narrow range (Table 1): 182.8 ± 1.7 nm (placebo nanoparticles) to 188.8 ± 1.7 nm (GRFT, DPV, or GRFT/DPV nanoparticles) and had low polydispersity index (PDI < 0.1), indicating that nearly monodispersed nanoparticles were manufactured (Figure 2). Zeta potential measurements showed minimal change between unloaded and loaded nanoparticles, falling within a range of −23.4 ± 0.3 mV to −24.9 ± 1.3 mV, respectively, at a pH of 7.4. The high similarity in colloidal properties of all nanoparticles such as size, PDI, and zeta potential demonstrates that the formed nanoparticles are composed of PLGA-PVA system. It is highly unlikely that the particles are primarily composed of GRFT alone. Table 1 lists properties of placebo nanoparticles (vehicle) and ARV-loaded nanoparticles fabricated using double emulsion evaporation.

A GRFT encapsulation efficiency of 40.7 ± 5.9% and 45.9 ± 13.7% was obtained from the manufactured single (NP-GRFT) and combination nanoparticles (NP-GRFT/DPV), respectively. A DPV encapsulation efficiency of 70.1 ± 4.4% and 69.4 ± 5.1% was obtained from NP-DPV and NP-GRFT/DPV, respectively. Our results indicate that the encapsulation efficiency of DPV and GRFT are independent of each other in the combined NPs. Our findings are similar to those describing the manufacture of PLGA nanoparticles via double-emulsion or solvent displacement methods [30,38,43]. Our results suggest that the double-emulsion technique is suitable for encapsulating both hydrophilic macromolecules and hydrophobic small molecules in PLGA-based core-shell type nanoparticles simultaneously.

### 3.2. In Vitro Release Studies of ARVs from PLGA Nanoparticles Resulting in Sustained Drug Release

**Figure 3:**
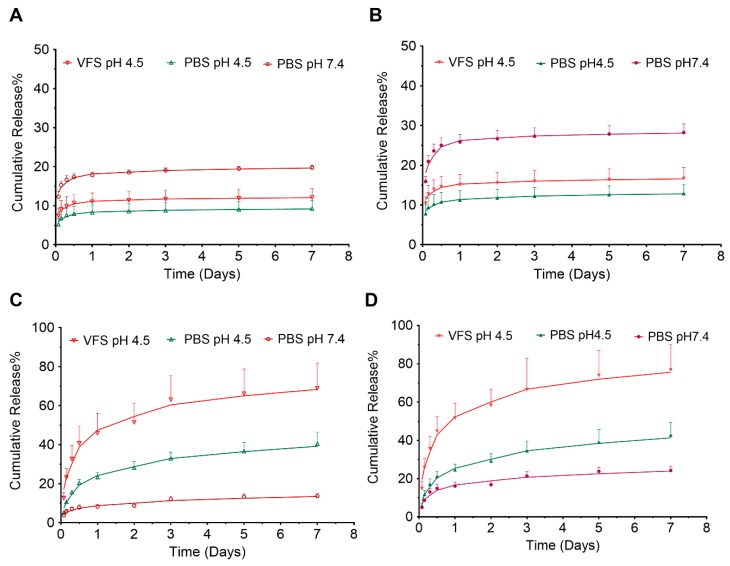
In vitro release of ARVs from PLGA nanoparticles in VFS at pH 4.5, and buffers at pH 4.5 and pH 7.4. (**A**) GRFT release from NP-GRFT and (**B**) NP-DPV/GRFT. (**C**) DPV release from NP-DPV and (**D**) NP-DPV/GRFT. NP-nanoparticles.

The in vitro drug release profile of GRFT and DPV from NP-ARVs was measured over 7 days in VFS at pH 4.5 that mimics the composition, pH, and viscoelastic properties of vaginal fluid produced by healthy, adult, nonpregnant women [48,59,60], as well as in buffers at pH 7.4 and pH 4.5. Our findings indicate that the combination of ARVs and media pH/composition affect the release profile of individual ARVs from core-shell PLGA nanoparticles as shown in Figure 3.

The in vitro release of GRFT and DPV from both single-entity and combination nanoparticles followed a biphasic release profile (Figure 3A,B). In VFS, the combination nanoparticles showed an initial burst release within first 24 h, where 15.1 ± 2.6% of the GRFT had been released. A sustained release of GRFT was obtained over the remaining 7 days. The total amount of drug released over the 7-day period was 16.7 ± 2.8%. Although not statistically significant, less GRFT was released from NP-GRFT, in both burst phase (11.1 ± 2.2%) and total amount of GRFT released (12.1 ± 2.8%) than that from combination nanoparticles.

The in vitro release of DPV showed a different and more prolonged initial burst release profiles (Figure 3C,D). In VFS, after 1 day, 51.7 ± 7.8% of DPV was released from the combination nanoparticles (NP-DPV/GRFT). DPV was released continuously over the next 7 days. The total amount of drug released over the 7-day period was 76.9 ± 13.1%. Similar to GRFT release, DPV release was lower from single entity nanoparticles in both burst phase (45.9 ± 10.0%), as well as the total amount of DPV released in 7 days (69.0 ± 12.8%) compared to combination nanoparticles in VFS and pH 7.4 media.

We also investigated the effect of pH and release media on the in vitro release of the PLGA nanoparticles. Total release of DPV from NP-DPV/GRFT was significantly higher in pH 4.5 media than that in pH 7.4 media (42.7 ± 6.7% vs. 24.3 ± 2.2%, *p* < 0.05). Even more DPV was released in VFS (pH 4.5) (76.9 ± 13.1%, *p* < 0.05) than in pH 4.5 media. However, pH showed an opposite effect on GRFT’s release from nanoparticles. The total release of GRFT from NP-DPV/GRFT was significantly less in pH 4.5 media than that in pH 7.4 (12.9 ± 2.2% vs. 28.2 ± 2.2%, *p* < 0.05), while the total release of GRFT in VFS (16.7 ± 2.8%) was only slightly higher than that in pH 4.5 media. The order of release in the media remained similar for the single entity nanoparticles (NP-DPV and NP-GRFT) compared to combination nanoparticles; however, stronger statistical differences were noted. The order of release rate of NP-ARVs is pH 7.4 > VFS > pH 4.5 for GRFT and VFS > pH 4.5 > pH 7.4 for DPV. These results suggest that co-encapsulation could lead to enhanced release of each ARV from PLGA nanoparticles in all the media investigated.

**Figure 4:**
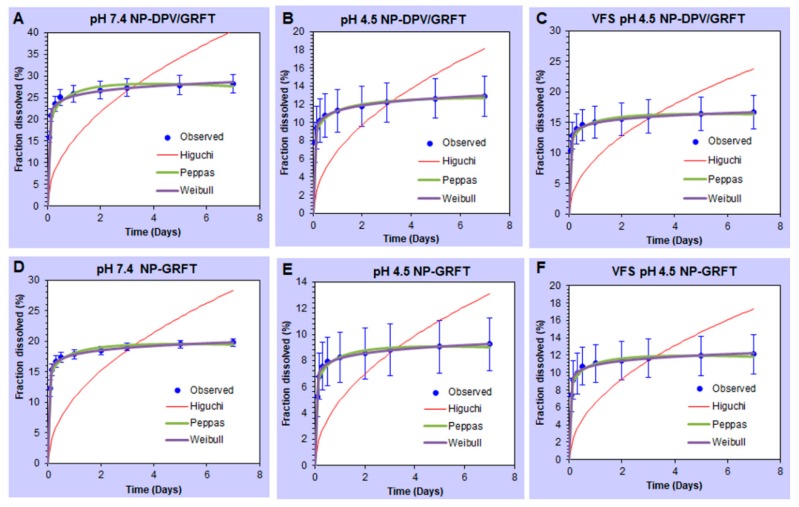
Mathematical model fitting of GRFT release from PLGA nanoparticles. (**A**–**C**) GRFT release from combination nanoparticles (NP-DPV/GRFT) at pH 7.4, 4.5 and VFS pH 4.5 media. (**D**–**F**) GRFT release from single entity nanoparticles (NP-GRFT) at pH 7.4, 4.5 and VFS pH 4.5 media. Dissolution profiles were fitted to Higuchi, Peppas, and Weibull models.

**Figure 5:**
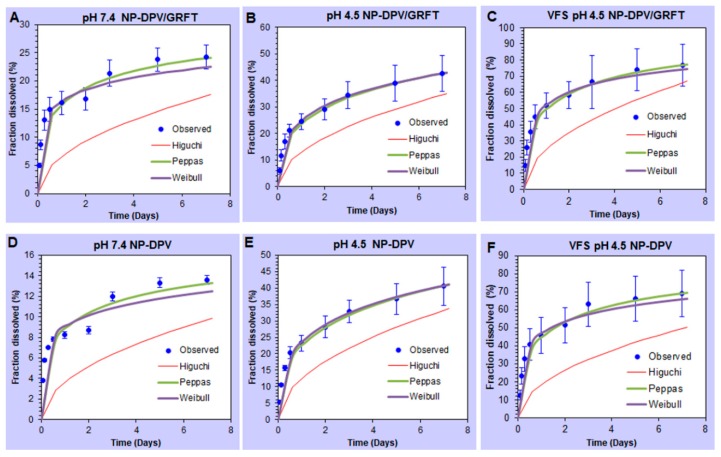
Mathematical model fitting of DPV release from PLGA nanoparticles. (**A**–**C**) DPV release from combination nanoparticles (NP-DPV/GRFT) at pH 7.4, 4.5 and VFS pH 4.5 media. (**D**–**F**) DPV release from single entity nanoparticles (NP-DPV) at pH 7.4, 4.5 and VFS pH 4.5 media. Dissolution profiles were fitted to Higuchi, Peppas, and Weibull models.

To reveal the mechanism of drug release from fabricated nanoparticles, we used mathematical modeling to analyze the in vitro release profiles of GRFT and DPV by various kinetic models. Three empirical and semi-empirical models used were the Higuchi model, the Peppas–Sahlin model, and the Weibull model [56]. Higher correlation was observed in the Peppas model and Weibull model (Figure 4 and Figure 5). Therefore, our results indicate that release of ARVs from PLGA nanoparticles is not predominantly driven by a solo mechanism, but a combined mechanism of Fickian (pure diffusion phenomenon) and non-Fickian release (due to the relaxation of the polymer chains between the networks).

### 3.3. PLGA NP-ARVs Potently Inhibit HIV-1_BaL_ Infection In Vitro

To ensure that the anti-HIV-1 activity of the ARVs was retained after being loaded into nanoparticles, we tested the protection of NP-ARVs and unformulated ARVs against HIV-1_BaL_ infection, using the TZM-bl assay. Anti-HIV-1 efficacy of NP-ARVs and unformulated ARVs was measured as percent of HIV inhibition, which is calculated using percent relative luminescence units of TZM-bl cells treated with ARVs or NP-ARVs compared to untreated (media only) TZM-bl cells after exposure to HIV-1_BaL_ as noted in the Methods section.

**Figure 6:**
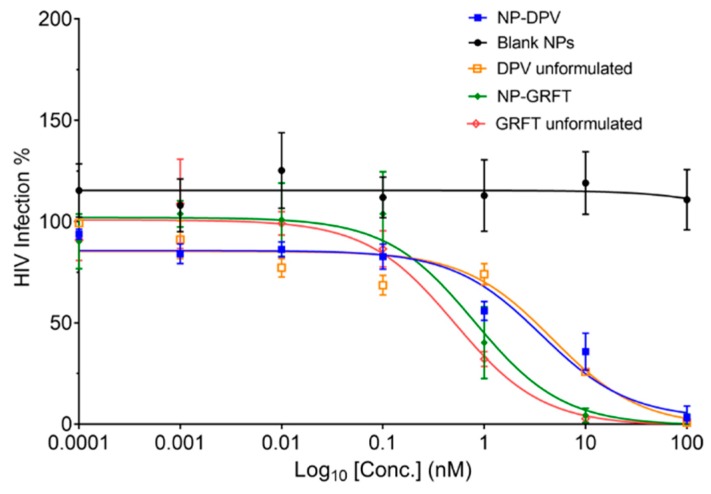
Anti-HIV activity of GRFT or DPV in PLGA nanoparticles. Anti-HIV activity of unformulated ARVs and fabricated NP-ARVs were evaluated in TZM-bl cells exposed to HIV-1. Luciferase luminescent readings of treated TZM-bl cells were compared against untreated cells infected with HIV-1 to obtain % HIV infection. Results are reported on a log scale of ARVs dosing levels versus percent of infection.

The anti-HIV-1 activity of blank PLGA nanoparticles was not observed in the TZM-bl assay, which was comparable to the negative media control. After exposure of TZM-bl cells to NP-ARVs or free ARVs; however, we observed potent antiviral activity against HIV-1_BaL_, with estimated IC_50_ values in the nanomolar ranges for GRFT and DPV, respectively (Table 2). Compared with free DPV, NP-DPV showed slightly enhanced HIV inhibitory activity, with an IC_50_ of 4.7 nM for the unformulated DPV to 3.6 nM for NP-DPV (Figure 6). The encapsulation of GRFT in PLGA nanoparticles slightly shifted the IC_50_ from 0.5 nM for the unformulated GRFT to 0.8 nM for the NP-GRFT. However, no statistically significant difference was observed between NP-GRFT and unformulated GRFT.

We hypothesized that NP-DPV can increase the intracellular concentration of DPV, resulting in greater potency due to enhanced internalization and intracellular uptake of PLGA nanoparticles [46,61]. For GRFT, whose target is on the cell membrane, the internalization and intracellular uptake of nanoparticles may cause the loss of bioactivity as we observed in the experiment, which is consistent with other reports on protein fusion inhibitors, such as RANTES [38]. However, no significant loss of GRFT’s bioactivity was observed. Together, our results suggest that both GRFT and DPV can be loaded into nanoparticles without compromising their potent bioactivity.

### 3.4. NP-ARV Combination Exhibits Strong Synergistic Bioactivity

To identify the interactive effects on our ARVs’ bioactivity, we evaluated the activity of their combination in nanoparticle and unformulated drugs, using the TZM-bl assay. The equipotency ratio of GRFT and DPV (i.e., 1:1 ratios of IC_50_ values), were used to assess the effect of the drug combination, when drugs were combined as unformulated or as distinct nanoparticles. This equipotency translated to a 1:8 molar ratio for GRFT:DPV. Notably, this exact ratio cannot be maintained in the manufactured combination nanoparticles (NP-DPV/GRFT), therefore these nanoparticles were tested at the loaded ARV levels.

**Figure 7:**
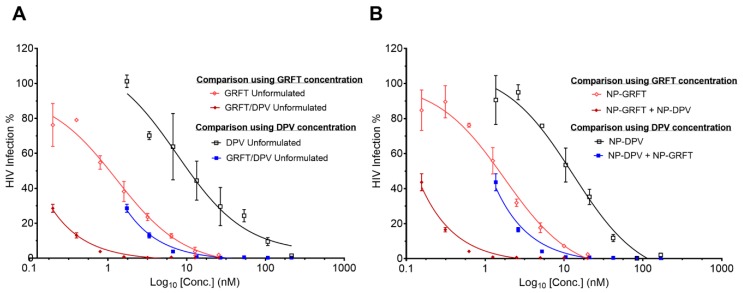
Strong synergistic effect of GRFT and DPV in free drug combination, in NP-GRFT and NP-DPV combination. The dose-response curve shows antiviral activity of (**A**) free GRFT, free DPV or combination of free GRFT and free DPV. (**B**) GRFT-loaded nanoparticles (NP-GRFT), DPV-loaded nanoparticles (NP-DPV) alone or in combination (NP-GRFT + NP-DPV). At a 1:8 molar ratio (GRFT and DPV), the antiviral activity of each drug in combination showed a significant reduction in %HIV infection (IC_50_ cannot be computed) compared to single ARV drug in unformulated or encapsulated forms. No difference in anti-HIV activity of GRFT or DPV were found in unformulated or nanoparticle combinations.

**Figure 8:**
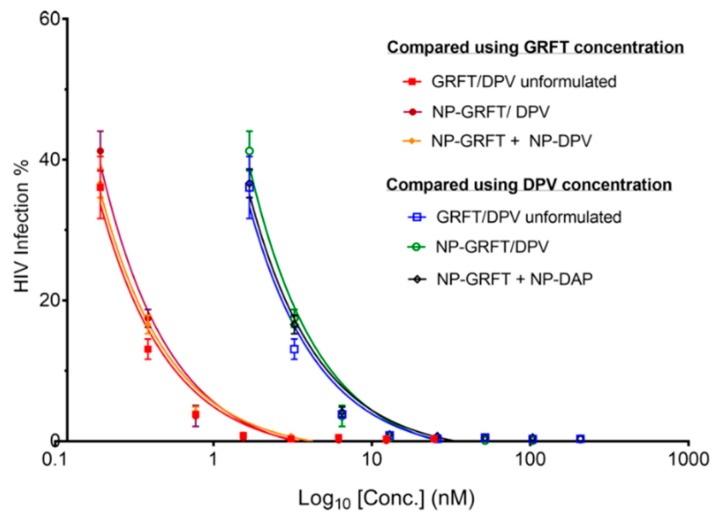
Potent and comparable anti-HIV effect of GRFT and DPV combinations. The dose-response curve shows potent antiviral activity of the combination of free drugs (GRFT/DPV unformulated), combination of single ARV nanoparticles (NP-GRFT + NP-DPV), and nanoparticles encapsulating both GRFT and DPV (NP-GRFT/DPV). Comparable inhibition of HIV infection was achieved with combination nanoparticles although the ARV ratio is not maintained at equipotency values (1:1 IC_50_ or 1:8 molar ratio for GRFT:DPV).

The efficacy of GRFT and DPV was significantly improved when tested in combination compared with when tested alone, either as unformulated GRFT and DPV or combined as distinct nanoparticles (NP-GRFT and NP-DPV) or when co-encapsulated into combination nanoparticles. Figure 7 shows the dose-response relationships of unformulated GRFT or DPV alone versus unformulated GRFT/DPV combination (Figure 7A), NP-GRFT or NP-DPV alone vs NP-GRFT and NP-DPV combination (Figure 7B). The results demonstrate that GRFT and DPV combined as unformulated or distinct nanoparticles or in a combination nanoparticle system display similar reduction in HIV infection. Interestingly, although the combination system does not include GRFT and DPV at equipotency ratio, a similar reduction in HIV infection was observed. IC_50_ values for combinations were not calculated, because even at the lowest tested concentration, high potency was observed, i.e., <50% HIV infection. Overall, the combination of GRFT and DPV showed significant leftward shift in the dose-response curve compared to single ARVs, indicating a strong synergy. The results also suggest that the efficacy is comparable between combination in unformulated, NP-GRFT and NP-DPV combination, and NP-GRFT/DPV, as shown in Figure 8.

**Figure 9:**
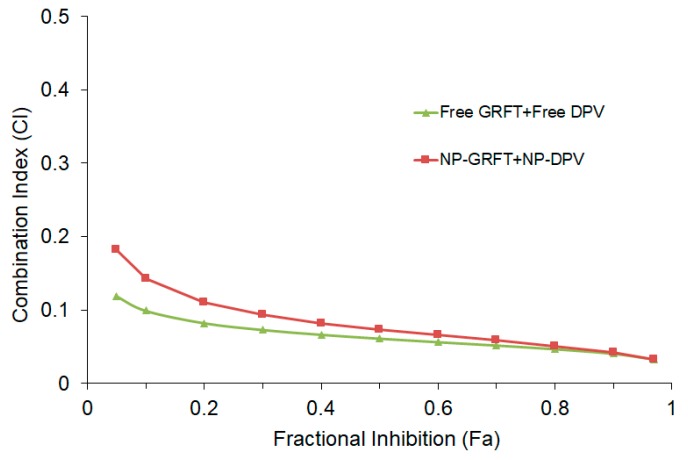
Combination index (CI) of free GRFT with free DPV or GRFT nanoparticles (NP-GRFT) with DPV nanoparticles (NP-DPV) were quantified using the TZM-bl infectivity assay. CI < 1, = 1, and >1 indicate synergistic, additive, and antagonistic effects, respectively. Combination of GRFT and DPV at a 1:8 molar ratio (GRFT:DPV) demonstrated very strong synergism, with CI at 50% inhibition (CI_50_) of 0.086 and 0.066 for unformulated and nanoparticle combinations respectively. CI 0.90–1.10: nearly additive; CI 0.85–0.95: slight synergism; CI 0.7–0.85: moderate synergism; CI 0.3–0.7: synergism; CI 0.1–0.3 strong synergism; CI < 0.1: Very strong synergism.

The CI was determined for the ARV drugs combined at molar ratios to achieve anti-HIV equipotency (1:1 ratios of IC_50_ values), leading to a 1:8 (GRFT: DPV) molar ratio in concentration. The CI of each unformulated ARV or NP-ARV combination was plotted as a function of the fractional inhibition (Fa) from 0.10 to 0.95 (Figure 9). The CI values were very low over the range from 0.10 to 0.95 of Fa. We interpreted the combination effects, at the CI_50_ and CI_90_ values, of unformulated GRFT/DPV and NP-GRFT/NP-DPV in combination (CI_50/90_ < 0.1) as demonstrating an extremely strong synergistic effect of anti-HIV infection.

### 3.5. NP-ARVs Are Nontoxic to In Vitro Cell Lines

**Figure 10:**
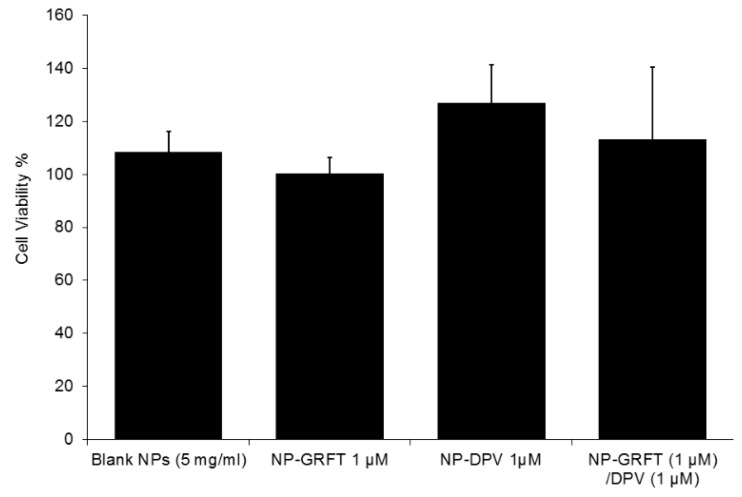
Toxicity of GRFT and DPV loaded PLGA nanoparticles. Viability of TZM-bl cells was measured by the CellTiter-Glo^®^ (Promega, Madison, WI, USA) viability assay kit demonstrating non-toxic nature (≥80% viability) of NP-GRFT, NP-DPV, and NP-GRFT/DPV. Vehicle control (blank nanoparticles) at the concentrations tested showed no reduction of viability (108% ± 7%), indicating non-cytotoxicity of PLGA polymer itself. Negative control = media only. Vehicle control for all nanoparticles was evaluated at 5.0 mg of polymer/mL.

Neither blank PLGA nanoparticles nor ARV-loaded PLGA nanoparticles were observed to be cytotoxic to cells over the range of concentrations evaluated in the TZM-bl assay. The cytotoxicity of NP-ARVs was evaluated during testing of their bioactivity in order to exclude any effects of the nanoparticles on the viability of TZM-bl cells. Cytotoxicity of our NP-ARVs was measured over a range of ARV concentrations, from 0.0001 to 1000 nM, after 24 h of exposure, leading to polymer concentration less than 0.1 mg/mL. No significant reduction (*p* < 0.05) in viability of the TZM-bl cells was observed in NP-ARV-treated cells as compared to untreated TZM-bl cells (Figure 10). In addition, we tried to evaluate the upper limit of cytotoxicity of nanoparticle vehicles. Compared with the negative control (media only), vehicle-control/blank NPs, at concentrations of 5 mg polymer/mL, showed no reduction of viability (100% ± 8%), suggesting that PLGA nanoparticles alone are not cytotoxic below this concentration. Since anti-HIV bioactivity was measured at doses far below 5 mg/mL polymer concentrations, we did not expect toxicity to confound the outcome of the antiviral activity assays.

### 3.6. Cellular Uptake of NPs In Vitro

**Figure 11:**
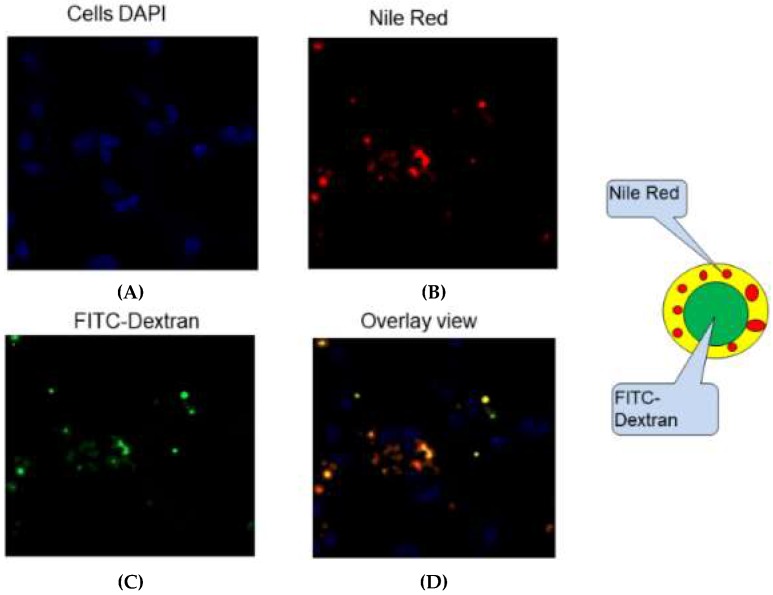
The cellular uptake of nanoparticles loaded with both FITC-Dextran (green, GRFT mimic) and Nile Red (red, DPV mimic) was investigated in RAW264.7 cells. Representative fluorescent microscopy images of RAW264.7 cells post nanoparticle exposure at 40× magnification. (**A**) Fluorescence images show RAW264.7 cells nuclei stained with DAPI (blue) for positioning purpose, (**B**) images of Nile red (red) and (**C**) FITC-dextran (green) of same field were taken through different channels to illustrate the position of FITC-dextran (green)/Nile red (red) encapsulated nanoparticles in cells. (**D**) Overlay image showing coincident green and red signals as yellow color indicating uptake of intact nanoparticles.

PLGA nanoparticles loaded with GRFT/DPV maintained the core-shell structure after cellular uptake. FITC-dextran and Nile red were incorporated into NPs as to evaluate the cellular uptake of NP-ARVs using fluorescence imaging as shown in Figure 11. FITC-dextran (Green) was used to mimic GRFT due to its water solubility and its molecular weight of 70 kDa. Nile red (Red) was used to mimic DPV due to its hydrophobicity. Coincident red and green fluorescence signals could be observed in the cytoplasm of RAW264.7 after 4 h incubation. The results demonstrate that the nanoparticles were taken up intact. Therefore, they could be delivered simultaneously in vivo with their synergistic anti-HIV effect.

## 4. Discussion

Combination and long-acting drug products are emerging approaches for the prevention of HIV-1 infection, due to the clinical need for effectiveness, safety, and patient adherence. To date, no combination PrEP is available, either as a vaginal or a rectal product, although efforts are underway. The only oral PrEP currently approved by the FDA (in 2004) is Truvada, which is a combination of emtricitabine and tenofovir disoproxil fumarate. Patient adherence plays a key role in the effectiveness of PrEP products [62], a lesson to be learned from several HIV prevention trials, which found that higher adherence resulted in lower HIV incidence [13,14,63]. Long-acting formulations offer a promising paradigm to overcome the adherence challenge in the development of PrEP products [64,65].

Two PrEP candidates have been intensively studied, individually, as potential microbicides in pre-clinical evaluations. GRFT, a fusion inhibitor, has demonstrated high efficacy against HIV-1 infection at nanomolar concentrations, with enhanced anti-HIV-1 activity and extended contact time with HIV-1 [18,20]. However, cervicovaginal secretions tend to inhibit GRFT-gp120 binding, as well as oxidize its methionine at position 78, resulting in compromise of GRFT in vivo [66]. DPV is an NNRTI with extreme potency against HIV-1 infection. Yet, its quick elimination from tissue may lower its anti-HIV-1 activity as a microbicide [23]. We hypothesized that core-shell polymeric nanocarriers could enhance the anti-HIV-1 effect of these two candidate ARVs via simultaneous co-delivery and sustained release for long-acting prevention of HIV-1 infection via vaginal route.

In this study, we demonstrated that a protein drug and a small hydrophobic drug can be encapsulated within polymeric nanoparticles to provide prophylaxis in combination. Anti-HIV-1 drugs were encapsulated into fabricated PLGA nanoparticles without compromising their anti-HIV-1 bioactivity. The nanoparticles were near-monodispersed, mostly spherical in shape. The constant negative zeta potential of the nanoparticles from the unloaded to the loaded state suggests that the hydrophilic microbicide is encapsulated in the core structure of nanoparticles rather than adsorbed onto the surface. High encapsulation efficiency of both GRFT and DPV suggests that the double emulsion method would be a viable approach for the fabrication of PLGA nanoparticles for other microbicide candidates.

We were able to demonstrate the sustained release of both GRFT and DPV, one of the defining characteristics of fabricated PLGA nanoparticles and indicating a potential long-acting nanoparticle delivery system. The in vitro drug release profiles of GRFT and DPV from the nanoparticles were characterized by two stages: the initial burst release followed by a sustained release over the time of the experiment. In vitro release studies showed that a significant amount of drug was released over a 7-day period, which may provide a weekly-based regimen.

The in vitro release of drugs from PLGA nanoparticles can be affected by environmental conditions such as changes in pH. Generally, PLGA polymer degrades faster at lower pH than at neutral pH [67,68]. The normal vaginal environment has an acidic pH, ranging from 3.8 to 4.5, as a protective barrier [69,70]. Therefore, in vitro release of drugs from PLGA nanoparticles was evaluated in buffers and VFS. Our studies showed that pH changes in the environment affected both the release rate and the total amount of GRFT and DPV released from the PLGA nanoparticles. Interestingly, we observed that pH change has opposite effects on the release of DPV and GRFT from the nanoparticles. More DPV was released into dissolution media from PLGA nanoparticles at lower pH (pH 4.5) than that at pH 7.4, which is expected due to the increased hydrolysis or erosion of PLGA polymers at lower pH [71]. Furthermore, DPV is likely to be in the protonated form at low pH, leading to increased solubility and release. Although both media possess similar pH, DPV release in VFS was much higher compared to the pH 4.5 buffer, possibly due to increased solubility of hydrophobic DPV in VFS, which contains bovine serum albumin. On the contrary, less GRFT was released from PLGA nanoparticles at lower pH (pH 4.5) than that at neutral pH (pH 7.4). One possible explanation for this phenomenon is that GRFT is positively charged at pH 4.5 due to its low isoelectric point (pI) of 5.39. Thus, the electrostatic interaction between positively charged GRFT and negatively charged PLGA nanoparticles may suppress the release of GRFT from PLGA nanoparticles. PSC-RANTES, for instance, has a much higher pI (around 9.0), retaining positive charge at both pH 7.4 and pH 4.5 [72]. Therefore, the release of PSC-RANTES is only determined by environmental pH [38]. Further studies are needed to confirm our hypothesis.

This compensatory release profile of our nanoparticles could prove beneficial for HIV-1 prevention. In a regular vaginal environment, more DPV will be released from the nanoparticles at lower pH (pH 4.5); when the presence of semen results in a higher pH environment (pH 7.4), more GRFT will be released. Our nanoparticle system could provide protection against HIV-1 infection in both scenarios. Therefore, it could be used in either a coitally dependent or coitally independent manner and provide protection over a wide range of environmental pH values.

Mathematical models were applied to evaluate the drug release from the nanoparticles. The results suggest that the release may be governed by a mixed mechanism of diffusion and polymer degradation, which is different from the polymer degradation–only mechanism of PSC-RANTES release from PLGA nanoparticles reported by Ham et al. [38]. However, the polymer chain relaxation may be the rate-limiting step for the drug release from the PLGA nanoparticle system. Further detailed studies are needed to elucidate the mechanisms involved, which is beyond the scope of this work.

Since the combination of GRFT and DPV is extremely potent, the HIV infection could not reach 50% in CI study, even at extremely low concentration of GRFT and DPV in combination, which made the estimation of IC_50_ for GRFT and DPV combination not very reliable. However, that also confirmed the potency of our combination strategy using GRFT and DPV. We found that the combination of free GRFT with free DPV at a 1:1 ratio of IC_50_ values demonstrated a strong synergistic effect (Figure 7). Importantly, the same synergistic effect was also demonstrated when the combination of drugs was released from nanoparticles, indicating that our PLGA nanoparticle system is suitable for delivering highly potent anti-HIV-1 drugs in combination. We quantified the synergistic effects of free GRFT and free DPV and NP-GRFT/NP-DPV using the median effect analysis developed by Chou and Talalay [73]. We believe that this synergy suggests a promising combination of ARVs as candidates for HIV-1 PrEP. Importantly, our studies demonstrated that GRFT and DPV possess a strong synergistic effect in vitro and that this synergistic effect can be maintained in PLGA nanoparticle delivery system. It had been reported that GRFT can have synergistic effects when used in combination with other anti-HIV-1 drugs, such as tenofovir [22]. However, it has never been studied in combination with DPV either as free drugs or in a nanoparticle system. Our findings indicate that the antiviral activity against HIV-1_BaL_ of our NP-ARVs was maintained similar to unformulated ARVs. The IC_50_ of DPV and GRFT did not change significantly in nanoparticles compared to unformulated form. Our results, however, show enhanced anti-HIV activity of NP-DPV (but *p* > 0.05), which may be caused by more uptake of nanoparticles [61]. The uptake of nanoparticles was visually confirmed in the RAW267.4 cell model. The combination NP-DPV/GRFT system also showed high anti-HIV-1 activity comparable to combination of unformulated ARVs and distinct NP-ARVs. Even without accomplishing 1:8 molar ratio of ARVs, our developed system displayed high potency and suitable for long-term application.

Interestingly, the potency of a drug may play an important role in the extent of combination effect with other drugs. Chaowanachan et al. reported that only additive effects were identified in a study of tenofovir and efavirenz delivery in combination in PLGA nanoparticles; this may be due to the limited drug release from the nanoparticles within the first hour [43]. Here, both tenofovir and efavirenz have an IC_50_ at a micromolar level, resulting in higher drug concentration needed for efficacy. Therefore, a faster drug release rate is required to achieve effective concentration against HIV infection. On the contrary, in our studies, both GRFT and DPV have an IC_50_ at a nanomolar level, indicating that only a small amount of the released drugs would be sufficient for complete HIV-1 inhibition. The difference between these studies using PLGA nanoparticles shows that the drug release rate needs to be optimized according to the drug potency by altering manufacturing and formulation conditions of the nanoparticles.

## 5. Conclusions

Although nanoparticles have been intensively investigated in numerous biomedical applications, not many studies have been conducted on nanoparticles as a drug delivery system for PrEP products, especially the combination delivery of a protein drug with a water-insoluble drug. In this study, successful encapsulation of both GRFT and DPV into core-shell nanoparticles resulted in monodispersed, near-spherical particles with low PDI that maintained their anti-HIV-1bioactivity. Furthermore, we have shown that our NP-ARVs act synergistically in preventing HIV-1 infection in vitro, and that the formulated PLGA nanoparticles provide sustained release of the drugs. To our knowledge, this is the first quantitative measure of synergistic effect of GRFT and DPV combination in a nanoparticle system. Additionally, the sustained release property of manufactured NPs provides a delivery system that could potentially reduce the dosing frequency to a weekly based regimen, which could significantly increase the compliance and acceptability in women.

However, additional research is required to further develop PLGA nanoparticles as PrEP products for delivery of combined GRFT and DPV. In particular, the relationship between protein pI and protein release in different media e.g., VFS. The model for drug release study in this paper only provides an empirical explanation on the mechanism of protein drug release from nanoparticles. Further investigation will help to reveal the mechanism of drug release from PLGA-based nanoparticles, particularly for protein drugs. In addition, the distribution of nanoparticles in the reproductive tract may affect the efficacy in vivo and needs to be investigated. These studies are beyond the scope of this paper and will be investigated in our future work.

## Figures and Tables

**Table 1:** Physicochemical properties of PLGA nanoparticles loaded with ARVs.

Drugs	Size (d.nm ± SD)	PDI	Zeta Potential (mV ± SD)	Encapsulation Efficiency (% ± SD)
Placebo	182.8 ± 1.7	0.066	−23.7 ± 0.6	-
GRFT	188.8 ± 1.7	0.069	−23.5 ± 0.3	40.7 ± 5.9
DPV	186.6 ± 1.6	0.079	−24.9 ±1.3	70.1 ± 4.4
GRFT/DPV	184.3 ± 1.0	0.063	−23.4 ± 0.3	45.9 ± 13.7 (GRFT) 69.4 ± 5.1 (DPV)

**Table 2:** IC_50_ of ARVs and NP-ARVs estimated by TZM-bl assay.

Drug	Alone (nM)	
	Unformulated	NP-GRFT/NP-DPV
DPV	4.7 ± 2.9	3.6 ± 2.9
GRFT	0.5 ± 0.3	0.8 ± 0.7
